# The relationship between parental locus of control and adolescent obesity: a longitudinal pre-birth cohort

**DOI:** 10.1038/s41366-018-0141-y

**Published:** 2018-07-09

**Authors:** Jean Golding, Yasmin Iles-Caven, Genette Ellis, Steven Gregory, Stephen Nowicki

**Affiliations:** 1 Centre for Child and Adolescent Health, Population Health Sciences, University of Bristol, Bristol, UK; 2 Department of Psychology, Emory University, Atlanta, GA USA

**Keywords:** Epidemiology, Risk factors

## Abstract

**Objectives:**

To investigate whether parental external locus of control (ELOC) measured in pregnancy is related to obesity in their adolescent offspring and whether the child’s own ELOC measured at age 8 contributes. To determine whether associations are due to types of behaviour used by externally oriented participants.

**Subjects/methods:**

Longitudinal pre-birth cohort study (Avon Longitudinal Study of Parents & Children (ALSPAC)) set in south-west England. Families whose adolescent offspring had their fat mass measured using DXA scans at any of ages 9, 11, 13, 15 or 17 (range, *n* = 7329 at 9 to *n* = 4850 at 17). The primary outcome measures were mean fat mass, and obesity measured as ≥85th centile of fat mass at each age.

**Results:**

We found that parent and child externality was associated with greater fat mass [e.g., mean difference at age 15 associated with maternal ELOC was 1.70 kg (+1.17, +2.24), paternal ELOC 1.49 kg (+0.89, +2.09) and child’s ELOC 1.50 kg (+0.93, +2.06) (*P* < 0.0001)]. Further analyses showed that factors associated with parent behaviour such as smoking in pregnancy, failure to breast feed, and early introduction of solids accounted for a third of the excess fat mass associated with maternal externality, whereas aspects of diet and energetic activity in later childhood were not. Further analyses demonstrated that the child’s own ELOC only became independently important for adolescent obesity from age 13, whereas the mothers’ and to a lesser extent the fathers’ ELOC were associated at each age.

**Conclusions:**

There is increased interest in determining factors that may be involved in the aetiology and maintenance of excessive weight in adolescents. We demonstrate that parental locus of control is a promising candidate. We suggest interventions to change parents’ locus of control towards internality in pregnancy might have long-term preventative benefits on the likelihood of obesity in the offspring.

## Introduction

The rate of obesity in childhood has been rising sharply across cultures throughout the world [1, 2]. It is estimated that about 20% of children and adolescents can now be classified as obese [3]. Tourtual [4] concludes that 'Overweight and obese children and adults represent an epidemic that threatens the quality and length of life (p. 17)'. Obesity has been related to greater incidences of cardiovascular disease [5], diabetes [6], high blood pressure [7] and cancer [8], as well as many negative psychological problems including increased anxiety [9], depression [10] and lower self-esteem and sense of well-being [11]. Obese children take their physical and emotional baggage with them into adulthood, where they show excessive morbidity and mortality rates [12].

Researchers, therefore, have attempted to identify possible ways in which the background of children, and of their parents, might be associated with excessive weight gain. One possible candidate is locus of control (LOC). Rotter [13] introduced the LOC concept within his social learning theory. He defined it as a generalised problem-solving expectancy which he explained as follows: 'Internal versus external control refers to the degree to which persons expect that a reinforcement or an outcome of their behaviour is contingent on their own behaviour or personal characteristics versus the degree to which persons expect that the reinforcement or outcome is a function of chance, luck, or fate, is under the control of powerful others, or is simply unpredictable.' His article stimulated a huge amount of research with over 17,000 studies published on this topic [14]. Findings with *internality* have been replicated across an impressive variety of psychological outcomes such as *improved* academic achievement [15, 16], sports performance [17] and business success [18, 19].

Parental externality could play a significant role in the way in which parents deal with their children. If parents are external there is an increased likelihood that they will be more likely to attribute their own health and well-being, and that of their children, to external forces beyond their control, and behave accordingly. This tends to result in a lack of structure for the developing child with consequent increases in poor behaviour [20, 21].

Researchers have found LOC to be relevant in successfully treating obesity in children [22]. Parent internal LOC has often been shown to be associated with successful weight loss [23, 24], but not always [25, 26]. The lack of a consistent LOC, obesity association may be due to the fact that most research has been carried out on small samples of children [27, 28], and used a variety of LOC scales making both generalisation and comparison of results difficult.

Here we take advantage of a large set of longitudinal data that allows for an extensive examination across ages, of the association between parental prenatal LOC, their offspring’s own LOC and subsequent measures of adolescents’ fat mass. Based on the theoretical description of LOC provided by Rotter [13] and others [29] and past empirical research, we make the general prediction that the greater the degree of prenatal parent and/or childhood externality, the greater the association with increased fat mass. The prediction is based on past findings supportive of the externality-obesity relation, as well as the association between parental externality and children’s negative eating and health behaviours [20]. In addition, theoretical assumptions derived from social learning theory would suggest that adult externality is associated with negative parenting behaviours such as impulsivity, inconsistency and less tendency to obtain advice/information. [13, 29–32]

## Material and methods

### The ALSPAC study

This pre-birth cohort was designed to determine the environmental and genetic factors that are associated with the health and development of children [33, 34]. As part of the study design, therefore, there was a concerted effort prior to the child’s birth to obtain from the parents, details of their personalities, moods and attitudes, including a measure of their LOC.

ALSPAC recruited 14,541 pregnant women resident in Avon, UK with expected dates of delivery between 1 April 1991 and 31 December 1992 (an estimated 80% of the eligible population). Data were collected at various time points using self-completion questionnaires, biological samples, hands-on measurements, and linkage to other data sets. For full details of all the data collected, through a fully searchable data dictionary, please see the study website: www.bristol.ac.uk/alspac/researchers/data-access/data-dictionary/. Ethical approval for the study was obtained from the ALSPAC Ethics and Law Committee and the Local Research Ethics Committees. Consent was implied if questionnaires were completed and returned. Informed consent was obtained for all invasive procedures.

For this project we concentrate on the parents’ LOC obtained from questionnaires completed prior to the index birth, and the child’s own LOC orientation collected at age 8. These are compared to the measures of fat mass of the adolescents as measured from ages 9 to 17.

#### The measures of locus of control

The LOC measure used in the present study is a shortened version of the adult Nowicki-Strickland Internal-External locus of control scale (ANSIE). This is described in detail elsewhere [21].

The children’s locus of control measure used in the present study was adapted from the Children’s Nowicki-Strickland Internal-External scale (CNSIE) [35] known as the Preschool and Primary Nowicki and Strickland Internal External control scale (PPNSIE). Both the CNSIE and PPNSIE have been used in over a thousand studies that have provided data supportive of their construct validity. The PPNSIE was administered to a sample of 120 8-year-old children and the 12 items with the best item-total correlation were chosen for inclusion in the final form administered to ALSPAC children at age 8. The questions were read aloud to the child by the examiner to eliminate variance due to reading ability. The child was asked to respond with a yes/no answer. The tester made clear that there were no right or wrong answers and the items reflected how people thought and felt about different things. The PPNSIE scores formed an approximately normal distribution with median of 6. As was the case for parents, externality was defined as having a LOC score above the median.

#### Measures of fat mass

A Lunar Prodigy narrow fan beam densitometer was used to perform a whole body DXA scan where bone, lean and fat masses are measured. The procedure was clearly explained to the child and parent, and both parental consent and child assent was obtained before proceeding. The child was asked to lie on the Prodigy couch (in light clothing without any metal fastenings), with the parent sitting at least a metre away to comply with the IRMER legislation. The arm of the machine moved over the child and two sources of X-ray scanned the child. The child was reassured throughout the scan and encouraged to keep as still as possible. The data from the DXA scan were used to calculate the fat mass in grams. Obesity was defined as on or above the 85th centile of the fat mass distribution.

#### Possible mediators

Since the development of poor growth on the one hand and overweight/obesity on the other is partly determined by diet, we tested the possibility that any association between parental prenatal LOC and childhood stunting or of overweight was due to differences in diets of children with internal compared to external parents. We considered three time points for the assessment of diet: in infancy, at age 3 and at age 10.

### Infant diet

Parents completed self-completion questionnaires at home about their child’s health and development at specific time points. The 4-week questionnaire collected data about breast and formula feeding for each week of the child’s life. At 6 months, the questionnaire included questions about breast and formula feeding, and frequency of consumption of specific foods and drinks. At 3 years of age a detailed food-frequency questionnaire was completed.

### Mid-childhood diet

At age 10, a 3-day dietary diary was sent to the parent a week before their child’s clinic appointment. They were asked to record everything the child ate or drank (including medicines) for a 3-day period (2 weekdays and 1 day at the weekend). Full instructions were included in the diary, with an example of a completed day, including how to record foods eaten at school.

The dietary records included detailed information about foods and drinks consumed, brands used and weights in household measures or taken from information given on the packaging of the food, as well as information about plate waste. At the clinic, a nutritionist checked the diaries with the child (accompanied by a parent) and missing or ambiguous details were clarified to improve accuracy and completeness. For 13.5% of children a diary had not been completed and a single 24-h recall was administered at the clinic.

Diet data were coded using DIDO software [36] and nutrient data were generated with an in-house programme using food composition information from the 5th edition of McCance and Widdowson’s food tables and supplements [37]. Portion sizes were coded using household measures and when these were missing a standard 10-year old child’s portion size was used based on data from the 1997 UK National Diet and Nutrition Survey [38]. For this study, as examples of nutrients that are assumed to be associated with obesity, we have used the estimates of the child’s intake of energy, trans-fatty acids and non-milk extrinsic sugars. These were arbitrarily chosen as nutrients known to be associated with obesity and/or risk of future ill-health: (i) non-milk extrinsic sugars (NMES) which are similar to free sugars but do not equate exactly. Free sugars are sugars added to foods by the manufacturer, cook or consumer, plus sugars present naturally in honey, syrups and unsweetened fruit juices. NMES also includes 50% of the fruit sugars from dried, stewed or canned fruit within the definition [39]. (ii) Trans-fatty acids which increase LDL and lower HDL cholesterol levels (they are reported to increase the risk of developing heart disease, stroke and type 2 diabetes) [40]. (iii) Energy which has to be taken into account when undertaking any dietary analysis, but which is also associated with increased weight.

### Activity levels

Others have shown, using ALSPAC, that adolescent fat mass is lower if the adolescent has been more active [41]. We hypothesised that such activity might be a means by which parental or child locus of control might influence the adolescent’s behaviour, in addition to dietary effects.

All ALSPAC children who attended the 11-year research clinic were asked to wear an Actigraph AM7164 2.2 accelerometer (Actigraph LLC, Fort Walton Beach, FL, USA) for 7 days. The mean age of the children was 11.8. The accelerometer was worn around the waist and detected acceleration and deceleration in a vertical plane as a combined function of movement frequency and intensity. Data were recorded as counts, averaged over a defined period (1 min in this study). The Actigraph has been validated in both children and adolescents. Children wore the accelerometer during waking hours, except for showering, bathing or any water sports. Data from children who had worn the accelerometer for at least 10 h/day for at least 3 days after deletion of missing data were considered valid, a level which we have previously shown achieves greatest power and good reliability [42]. Two variables were used—total physical activity and time spent in moderate to vigorous physical activity (usually defined as any activity at least equivalent to the physiological stress of brisk walking for a person of average fitness).

### Statistical analyses

Our aim was to determine the degree to which maternal, paternal and childhood external LOC orientations influence the development of obesity (as measured by (i) the amount of fat mass, and (ii) fat mass ≥85th centile). The analyses undertaken use: (i) multiple regression for the outcome mean fat mass (kg) and (ii) logistic regression for the binary outcome of obesity. Because of collinearity between maternal and paternal LOC we did not put both into the analysis but rather carried out stepwise analyses to determine which were independent of one another. The externality of each individual was taken as the exposure. The first analyses (model A) allowed just for sex and ethnicity of the child. These analyses were considered the basic results. Model B assessed whether the associations were ‘explained’ by behavioural factors: maternal smoking in pregnancy, duration of breast feeding the child and age at which solids were introduced, each of which has been shown to be related to offspring obesity [43–45]. Model C then added the dietary and activity measures as measured at 10 and 11.

Analyses were also carried out to determine whether there were sex differences in the effect sizes between the two sexes. For these analyses, measures of pubertal status were also included in model A, using self-reported assessments using illustrations of Tanner stage of pubic hair development for boys and breast development for girls. They were completed by the participant and/or a parent in specially designed postal questionnaires in their own homes [46, 47].

## Results

### Fat mass measurements

The basic information concerning the mean and the 85th centile of the fat mass for each age group of the adolescents is shown in Supplementary Table 1. This shows that: (i) the numbers of individuals measured reduce over time from 7329 at age 9 to 4850 at age 17; this is not unexpected since attrition is a function of all current longitudinal studies. (ii) Mean fat mass increases steadily from 8.58 kg at age 9 to 18.31 kg at age 17. (iii) The level of the 85th centile also increases rapidly over time—from 13.76 to 28.15 kg over the 8 years.

#### Unadjusted mean fat mass and parental LOC

The adolescents’ mean fat mass was considerably higher at all ages if the mother or father was externally oriented prenatally (Supplementary Table 2 and Table 1). This was true for each age of the offspring, and increased over time, the difference being greater than 1.5 kg by age 17.

**Table 1:** Difference between mean fat mass (kg) [95% CI] of adolescents comparing the external with the internal orientation of the parents and of the child at age 8

Age of adolescent	Maternal LOC	Paternal LOC	Child’s LOC at 8 years
9 years	+0.71 [+0.46,+0.96]*	+0.58 [+0.30,+0.86]*	+0.16 [−0.10,+0.43]^NS^
11 years	+0.91 [+0.56,+1.25]*	+0.83 [+0.45,+1.22]*	+0.46 [+0.10,+0.83]**
13 years	+1.37 [+0.93,+1.80]*	+1.27 [+0.79,+1.75]*	+1.15 [+0.69,+1.61]*
15 years	+1.70 [+1.17,+2.24]*	+1.49 [+0.89,+2.09]*	+1.50 [+0.93,+2.06]*
17 years	+1.57 [+0.93,+2.22]*	+1.60 [+0.88,+2.31]*	+1.76 [+1.09,+2.44]*

*LOC* Locus of control

*P < 0.0001; **P < 0.05; ^NS^*P* > 0.05

Within pairs of parents the variation in mean fat mass differed with the highest fat mass occurring if both parents were external, and the lowest if both were internal (Table 2); if only one of the parents was external, the mean fat mass was between the two extremes, but there was little indication that the offspring of an external mother/internal father differed from that of an internal mother/external father. Thus, the externality of each parent appeared to have independent effects. We have therefore analysed them separately.

**Table 2:** Mean fat mass (kg) of children of external mothers comparing those whose partners are also external with those whose partners are internal, and children of internal mothers comparing those whose partners are external with those who are internal

Age	Both external	Only mother external	*P* ^a^	Only father external	Both internal	*P* ^b^
9 years	9.08	8.82	NS	8.57	8.03	**
11 years	12.46	11.99	NS	11.74	11.02	**
13 years	14.88	14.06	(*)	13.85	12.72	***
15 years	16.82	15.89	(*)	15.54	14.25	***
17 years	19.68	18.81	NS	18.55	16.99	***

^NS^*P* > 0.10; ^(*)^*P* < 0.10; **P < 0.01; ***P < 0.001

^a^*P* comparison of the first two columns

^b^*P* comparison of next two columns

#### Adjusted mean fat mass and parental LOC

Table 3 shows the excess mean fat mass if the mother was external: at each age the adolescent has substantially more fat than the offspring of an internal mother after allowing for ethnicity and sex of the child. Additional analysis assessed whether the external parent, child obesity relationship was mediated by the early behaviour of the mother (smoking in pregnancy, duration of breast feeding and age at introduction of solids; model B). This indicated that these factors accounted for about a third of the excess fat of an adolescent offspring of an external mother; the reductions at each age were about 220, 330, 440, 370 and 630 g, respectively. In contrast, further analyses (model C) indicated that diet and activity levels at age 10/11 did not reduce further the effect size of the maternal externality association, indicating that these later factors were not additional mediators. Similar but less impressive results were found for the father’s externality in pregnancy (Supplementary Table 3), with reductions of 150, 270, 345, 258 and 400 g for model B compared with model A, and little further reduction for model C.

**Table 3:** Associations between maternal external locus of control as measured in pregnancy and fat mass in adolescence

Age of offspring	Model A	Model B	Model C
9 years			
MD [95% CI]	0.66 [0.42, 0.91]	0.44 [0.18, 0.70]	0.44 [0.14, 0.73]
*P*	<0.0001	0.001	0.004
*N*	6443	6071	4270
11 years			
MD [95% CI]	0.89 [0.55, 1.23]	0.56 [0.20, 0.91]	0.64 [0.26, 1.03]
*P*	<0.0001	0.002	0.001
*N*	6183	5828	4573
13 years			
MD [95% CI]	1.18 [0.77, 1.58]	0.73 [0.31, 1.16]	0.90 [0.43, 1.37]
*P*	<0.0001	0.001	<0.001
*N*	5378	5110	3952
15 years			
MD [95% CI]	1.30 [0.82, 1.78]	0.93 [0.43, 1.44]	0.98 [0.42, 1.53]
*P*	<0.001	<0.001	0.001
*N*	4608	4369	3449
17 years			
MD [95% CI]	1.26 [0.68, 1.85]	0.63 [0.01, 1.25]	0.70 [0.02, 1.39]
*P*	<0.001	0.045	0.045
*N*	4262	4043	3055

The table shows the mean difference [95% CI] (kg) in fat mass for offspring of externally compared with internally oriented women

Model A allows for sex and ethnicity of the child; model B additionally allows for duration of breast feeding, age started solids and whether the mother smoked at mid-pregnancy; model C is model B plus aspects of the offspring diet at ages 10 (energy, trans-fatty acids and sugar) and activity levels at 11

*MD* mean difference, *CI* confidence interval

#### Sex differences between parental LOC and adolescent fat mass

The basic relationship between maternal ELOC and fat mass (model A) was stronger for girls than for boys (Supplementary Table 4), and this difference remained after allowance for possible mediators (model C). Differences between girls and boys were nonsignificant for paternal ELOC whether comparing model A or model C (Supplementary Table 5).

#### Associations between the child’s ELOC and adolescent fat mass

The child’s own LOC was obtained at age 8. Subsequent measures of fat mass as adolescence progressed (Table 4) indicated that externality at age 8 was not significantly associated with fat mass at 9, only moderately associated at age 11, but significantly higher at the older ages. Possible mediators did not account for the excess fat mass associated with the child’s measure of externality at any age. There were no differences between boys and girls in this regard (Supplementary Table 6).

**Table 4:** Associations between the child’s external locus of control as measured at age 8 and fat mass in adolescence

	Model A	Model B	Model C
9 years			
MD [95% CI]	0.14 [−0.12, 0.40]	0.05 [−0.22, 0.33]	0.14 [−0.17, 0.45]
*P*	0.301	0.708	0.386
*N*	5463	5160	3767
11 years			
MD [95% CI]	0.46 [0.09, 0.83]	0.33 [−0.05, 0.71]	0.37 [−0.04, 0.78]
*P*	0.015	0.092	0.075
*N*	5167	4886	3961
13 years			
MD [95% CI]	1.12 [0.67, 1.57]	0.93 [0.46, 1.39]	0.81 [0.31, 1.31]
*P*	<0.0001	<0.0001	0.002
*N*	4576	4352	3488
15 years			
MD [95% CI]	1.37 [0.84, 1.89]	1.23 [0.68, 1.77]	1.14 [0.54, 1.74]
*P*	<0.0001	<0.0001	<0.0001
*N*	3975	3777	3067
17 years			
MD [95% CI]	1.42 [0.77, 2.07]	1.24 [0.57, 1.91]	1.20 [0.47, 1.94]
*P*	<0.0001	<0.0001	0.001
*N*	3548	3450	2713

The table shows the mean difference [95%CI] (kg) in fat mass for externally compared with internally oriented 8-year olds

Model A allows for sex and ethnic background of the child; model B additionally allows for duration of breast feeding, age started solids and whether the mother smoked at mid-pregnancy; model C is model B plus aspects of the offspring diet at ages 10 (energy, trans-fatty acids and sugar) and activity levels at 11

*MD* mean difference, *CI* confidence interval

#### Obesity and LOC

**Fig. 1:**
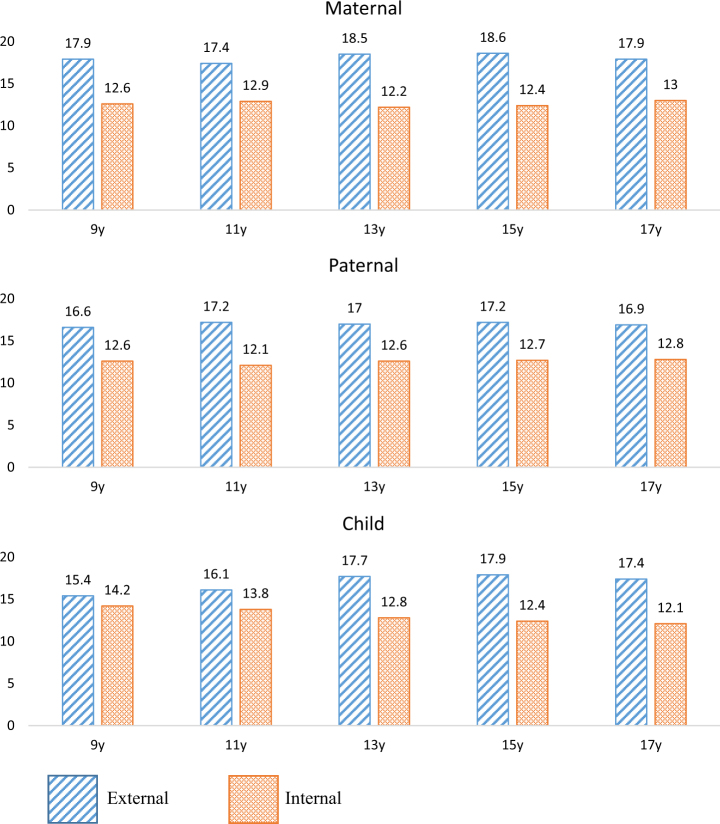
The percentage of adolescents with a fat mass ≥85th centile at different ages according to whether the mother, father or child was external or internal

The proportion of obese adolescents at each age (defined as having a fat mass ≥85th centile), is significantly higher (*P* < 0.0001) if the parent had indicated that he/she had an external orientation during pregnancy (Fig. 1). In contrast, the proportion of obese children at age 9 did not vary with their own LOC orientation measured at age 8. However, as the children got older, the contrast between the proportion of children who were obese became more marked and more statistically significant when their own orientation was external.

Further analysis indicated that at ages 9 and 11 the parental externality association with obesity predominated, but that from age 13 onwards, the child’s externality was independently associated with obesity, and was not a consequence of the parents’ LOC orientation (Table 5). However, the parents’ LOC orientation (particularly the mother’s) was strongly associated with offspring obesity independent of the child’s orientation, even at age 17. Further analyses assessing the risk of obesity with each of the three LOC measures of externality and the models A, B and C are shown in Supplementary Table 7. As with the analyses of mean fat mass, there is an indication that part of the excess risk to adolescent offspring of external mothers concerns the early health behaviour of the mother, but no consistent reductions associated with later childhood diet or exercise.

**Table 5:** The result of stepwise logistic regression offering the three measures of locus of control

Age	Group	Mother’s ELOCn or [95% CI]	Father’s ELOC or [95% CI]	Child’s ELOC or [95% CI]	*N*	GOF
9	All	1.46 [1.24,1.73]*	1.26 [1.07,1.48]**	DNE	4965	0.85
11	All	1.44 [1.22,1.70]*	1.40 [1.18,1.65]*	DNE	4789	1.08
13	All	1.67 [1.37,2.04]*	1.26 [1.03,1.53]***	1.29 [1.06,1.57]**	3558	1.68
15	All	1.62 [1.31,2.00]*	1.24 [1.00,1.53]***	1.31 [1.07,1.62]**	3127	1.56
17	All	1.33 [1.09,1.62]**	DNE	1.47 [1.21,1.78]*	3589	0.89

Outcome = obesity measured as ≥85% fat mass

*DNE* did not enter, *ELOC* external locus of control, *GOF* goodness of fit measures estimated from pseudo R^2^, *N* no. of adolescents in final model

*P < 0.0001; **P < 0.01; ***P < 0.05

## Discussion

This set of analyses was devised to assess whether, and to what extent, the external orientation of the mothers and fathers, when measured during pregnancy, was associated with increased fat mass in their adolescent offspring, and whether the child’s own externality was associated with obesity. We found that: (a) external orientations of each parent measured in pregnancy was associated with increased fat mass in the adolescents; (b) the child’s externality measured at age 8 was strongly associated, but only from age 13 onwards; (c) the mothers’ externality association with offspring fat mass was partly mediated by her smoking during pregnancy, failing to breast feed and starting the child on solids earlier than recommended; (d) diet and activity of the child at age 10 did not explain further any of the associations of parents’ or child’s externality with mean fat mass or obesity.

In this study a limited number of parenting and dietary variables were evaluated for their possible role in the external parental LOC and obesity association. They were chosen because past research [41, 43–45] had shown that they were related to both external locus of control as well as to obesity. Though they accounted for a third of the excess fat mass associated with parental ELOC, two-thirds of the excess fat mass remained unidentified. At age 10, we arbitrarily chose three nutrients known to be associated with obesity and/or risk of future ill-health: non-milk extrinsic sugars, trans-fatty acids and energy. We failed to show that these indicators at age 10 explained any of the remaining associations between ELOC and adolescent fat mass. Nor was the activity level of the child at age 11 able to explain the effects although this was clearly relevant to the adolescents’ fat mass [41]. It is important to note that this appears to be among the first studies to have been able to show a time relationship. Although there is much evidence cross-sectionally of a correlation between externality and obesity [48], it was not obvious as to which came first—in other words did the externality develop in response to becoming obese or vice versa. We have been able to show that the LOC orientation of the child precedes the development of obesity by at least 5 years.

Further research is needed to evaluate the possible contribution of other characteristics of the families. We did not include features such as parental educational attainment as it is well documented that educational achievements are associated with, and likely to be a consequence of, externality in the parents’ own childhoods; inclusion in the analyses are likely to result in an over-control. The present study identified factors from early, rather than later, in childhood as important in explaining some of the ELOC obesity association. However, other later childhood factors not yet evaluated might prove to be significant, particularly in explaining the excess risk of obesity associated with the child’s own ELOC. As well as other components of the diet at different ages, candidates include: (a) Sleeping habits: external parents are more likely to have children with sleeping problems [20], and others have demonstrated that short duration of sleep at age 3 is associated with later obesity [49]. (b) Genetics: there is substantial evidence that obesity clusters in families, and that some of this is associated with inherited genes (e.g., the FTO gene [50]). However, there is no evidence that these genes are associated with LOC orientation. (c) Puberty: transition to puberty is associated with changes in body composition, and girls in particular have markedly increased fat mass. When assessing the differences between the sexes, we allowed for pubertal stage using the age at which pubertal hair started appearing in boys and breast development in girls. Although it was important to take these stages into account, we found no evidence that the LOC of either the parents or the individual was associated with precocious or retarded puberty.

Parental ELOC assessed before the child was born was associated with long-term increases in body fat, which may be partly explained by choices external parents make raising their offspring. While the present design does not establish causality, the results suggest that it might be fruitful to examine the impact of programmes to change prenatal locus of control towards internality on children’s obesity. Programmes to change the child’s ELOC could also have long-term benefits if the relationship is causal. Not only have we shown that having an ELOC is associated with obesity in teenagers, but an earlier UK cohort of births in 1970 found that an external LOC at age 10 was associated with an increase in obesity at age 30 [51].

The strengths of this project lie in: (a) the use of a prospective design; (b) the use of a large and representative population of participants; (c) the inclusion of mother’s, father’s and child’s LOC; (d) objective measures of fat mass using DXA scans, which give a more focussed measure of obesity than body mass index (BMI), which includes lean mass such as muscle; (e) the LOC orientation of each parent was obtained before the child was born, and was therefore not influenced by the characteristics of the child.

However, there are a number of limitations: (i) the analyses were restricted to the 80% of the eligible pregnant population that took part in the study. The mothers who did not take part were biased in that they were somewhat more likely to be teenagers and/or of low educational achievement (features that have been found associated with externality). In spite of this it has been shown that the demographic differences between the mothers who enroled and those who did not were relatively small [52]. (ii) To date, there have been no studies that have collected data to test whether the same associations are apparent in different communities.

In conclusion, there is increasing evidence that parent–child interactions during the early years (including the prenatal period) are important for the health and development of children later. In light of previous research, findings highlighting the connections between LOC and personal, social and health outcomes, it is not surprising that we have found such strong associations between prenatal parental ELOC and obesity in their adolescent offspring. What had not been found before, however, was the significant and continuing association between parent prenatal externality and adolescent obesity.

Anzman and colleagues [53] have argued that the first years of life, including the prenatal period, the postnatal suckling period and the transition to the modified adult diet, may provide opportunities for preventive interventions. They point out that these early periods are characterised by high plasticity and rapid transitions, and parents have a high degree of control over children’s environments and experiences. They go on to suggest that interventions to change parenting behaviours in regard to diet in the early months of life may be particularly productive. Pertinent to the findings in the present study, previous studies have found parent externality to be associated with negative outcomes in children’s behaviour from 4 weeks to early adolescence [20, 21]. In addition, parent prenatal externality was associated with reduced likelihood of their attending prenatal classes in which diet and parenting techniques were discussed. As a result, external parents may lack the necessary education concerning child care; this, coupled with their belief that there is not much of a connection between how they behave as parents and outcomes of their children, may result in them being less prepared to effectively care for or teach their children healthy ways of behaving. Campis and colleagues [54] agree and suggest that parents with external locus of control orientations possess several negative concomitant attitudes about their parental roles such as low self-efficacy and a sense of being dominated by their child’s demands that hamper their effective parenting.

One potential use of this information is to develop programmes that would either change the locus of control of parents towards internality or provide prenatal and child rearing information more attuned to the way externals learn, to see if children would be more likely to develop healthy behaviours and be less likely to become obese. In support of the utility of this approach, we know from randomised controlled trials of ill patients that training participants to become more internal results in better compliance with recommendations and improved outcomes [55]. Both Hagekull et al. [56] and Moreland et al. [57] have found that changes in parent locus of control towards internality were associated with positive changes in children’s social adjustment; it is possible that changing parents’ prenatal locus of control towards internality may have a positive effect on children’s outcomesincluding obesity.

Programmes do exist for producing greater internality in adults. A key component of the programmes is increasing awareness of externality in speech and behaviour and providing internal alternatives [58]. Wolinsky et al. [59] added a more formal 'cognitive training experience' to awareness training that included a structured set of learning experiences clearly emphasising connections that did and did not exist between adult behaviours and outcomes. Nowicki [60] offered a self-guided programme to develop appropriate internality in adults that emphasised: (1) greater awareness of outcomes of behaviour, (2) setting realistic goals, (3) taking responsibility for both positive and negative behavioural outcomes, (4) evaluating daily choices and (5) using the result of the process to create realistic expectancies regarding future outcomes.

Parent internality has been associated with an impressive set of child outcomes in the past. More recently, it has become apparent that prenatal parent externality also can produce associations with child outcomes very early in children’s lives and continuing throughout childhood. Although the design of the present study did not allow for the establishment of causality, the prospective findings suggest that there might be payoff in evaluating the possible association between helping prenatal parents to become more internal and child outcomes including obesity [61].

## Electronic supplementary material

Supplementary Tables

## References

[1] Freedman DS, Khan LK, Serdula MK, Ogden CL, Dietz WH (2006). Racial and ethnic differences in secular trends for childhood BMI, weight, and height. Obesity.

[2] Ng M, Fleming T, Robinson M, Thomson B, Graetz N, Margono C (2014). Global, regional, and national prevalence of overweight and obesity in children and adults during 1980–2013: a systematic analysis for the Global Burden of Disease Study 2013. Lancet.

[3] Ogden CL, Carroll MD, Kit BK, Flegal KM (2014). Prevalence of childhood and adult obesity in the United States, 2011-2012. J Am Med Assoc.

[4] Tourtual JM (2014). The relationships of parental self-efficacy, parental locus of control, children’s health-promoting behaviors, and childhood obesity.

[5] Dietz WH (1998). Health consequences of obesity in youth: childhood predictors of adult disease. Pediatr Mar.

[6] Al-Goblan AS, Al-Alfi MA, Khan MZ (2014). Mechanism linking diabetes mellitus and obesity. Diabetes Metab Syndr Obes.

[7] Falkner B, Gidding S (2011). Childhood obesity and blood pressure: back to the future?. Hypertension.

[8] De Pergola G, Silvestris F (2013). Obesity as a major risk factor for cancer. J Obes.

[9] Esposito M, Gallai B, Roccella M, Marotta R, Lavano F, Lavano SM (2014). Anxiety and depression levels in prepubertal obese children: a case-control study. Neuropsychiatr Dis Treat.

[10] Reeves GM, Postolache TT, Snitker S (2008). Childhood obesity and depression: connection between these growing problems in growing children. Int J Child Health Hum Dev.

[11] Wilson AL, Goldfield GS (2014). Overweight or obese young people are not at increased risk of depression, but young people with depression are at increased risk of obesity. Evid Based Nurs.

[12] Reilly JJ, Methven E, McDowell ZC, Hacking B, Alexander D, Stewart L (2003). Health consequences of obesity. Arch Dis Child.

[13] Rotter J (1966). Generalized expectancies for internal versus external control of reinforcement. Psychol Monogr.

[14] Nowicki S, Duke MP, Infurna F, Reich JW (2016). Foundations of locus of control research. Perceived control: theory, research, and practice in the first 50 years.

[15] Flouri E (2006). Parental interest in children’s education, children’s self-esteem and locus of control, and later educational attainment: twenty-six year follow-up of the 1970 British Birth Cohort. Br J Educ Psychol.

[16] Kalechstein A, Nowicki S (1997). A meta-analytic examination of the relationship between control expectancies and academic achievement: an 11-year follow-up to Findley and Cooper. Genet Soc Gen Psychol Monogr.

[17] Arnaud J, Palazzolo J (2012). Link between locus of control and competitive anxiety: study of 150 high-level tennis players. Ann Med Psychol.

[18] Spector P, Cooper CL, Sanchez JI (2002). Locus of control and well-being at work: how generalizable are western findings?. Acad Manag J.

[19] Wu CH, Griffin MA, Parker SK (2015). Developing agency through good work: longitudinal effects of job autonomy and skill utilization on locus of control. J Vocat Behav.

[20] Nowicki S, Iles-Caven Y, Gregory S, Ellis G, Golding J (2017). The impact of prenatal parental locus of control on children’s psychological outcomes in infancy and early childhood: A prospective 5-year study. Front Psychol.

[21] Nowicki S, Gregory S, Ellis G, Iles-Caven Y, Golding J (2018). Parental external locus of control in pregnancy is associated with subsequent teacher ratings of negative behaviour in primary school: findings from a British birth cohort. Front Psychol.

[22] Rosno EA, Steele RG, Johnston CA, Aylward BS (2008). Parental locus of control: associations to adherence and outcomes in the treatment of pediatric overweight. Child Health Care.

[23] Turner TT (2017). Parental locus of control relative to body image and child outcomes. A dissertation.

[24] Springer NS, Bogue EL, Arnold M, Yankou D, Oakley D (1994). Nutrition locus of control and dietary behaviors of pregnant women. Appl Nurs Res.

[25] Favaro A, Santonastaso P (1995). Effects of parents’ psychological characteristics and eating behavior on childhood obesity and dietary compliance. J Psychosom Res.

[26] O’Dea JA, Wilson R (2006). Socio-cognitive and nutritional factors associated with body mass index in children and adolescents: possibilities for childhood obesity prevention. Health Educ Res.

[27] Geller SE, Keane TM, Scheirer JC (1981). Delay of gratification, locus of control, and eating patterns in obese and nonobese children. Addict Behav.

[28] Moss ND, Dadds MR (1991). Body weight attributions and eating self-efficacy in adolescence. Addict Behav.

[29] Lefcourt HM (1976). Locus of control: current trends in theory and research.

[30] Lefcourt HM (1982). Locus of control: current trends in theory and research. 2nd edn.

[31] Rotter J (1975). Some problems and misconceptions related to the construct of internal versus external control of reinforcement. J Consult Clin Psychol.

[32] Rotter J (1990). Internal versus external control of reinforcement: a case history of a variable. Am Psychol.

[33] Golding J (2004). The Avon Longitudinal Study of Parents and Children (ALSPAC) – study design and collaborative opportunities. Eur J Endocrinol.

[34] Boyd A, Golding J, Macleod J (2013). Cohort profile: the ‘children of the 90s’—the index offspring of the Avon Longitudinal Study of Parents and Children. Int J Epidemiol.

[35] Nowicki S, Strickland BR (1973). A locus of control scale for children. J Consult Clin Psychol.

[36] Price GM, Paul AA, Key FB (1995). Measurement of diet in a large national survey: comparison of computerised and manual coding of records in household measures. J Hum Nutr.

[37] Holland B, Welch AA, Unwin ID (1991). McCance and Widdowson’s the composition of foods.

[38] Gregory J, Lowe S (2000). National Diet and Nutritional Survey: young people aged 4 to 18 years. Vol 1: Report of the diet and nutrition survey.

[39] British Nutrition Foundation. https://www.nutrition.org.uk/nutritioninthenews/headlines/letstakeanotherlookatsugar.html.. Accessed 27 December 2017.

[40] American Heart Association. https://healthyforgood.heart.org/eat-smart/articles/trans-fat. Accessed 1 February 2018.

[41] Riddoch CJ, Leary SD, Ness AR, Blair SN, Deere K, Mattocks C (2009). Prospective associations between objective measures of physical activity and fat mass in 12-14 year old children: the Avon Longitudinal Study of Parents and Children (ALSPAC). Br Med J.

[42] Riddoch C, Edwards D, Page A, Froberg K, Anderssen SA, Wedderkopp N (2005). The European Youth Heart Study—cardiovascular disease risk factors in children: rationale, aims, study design, and validation of methods. J Phys Act Health.

[43] Oken E, Levitan EB, Gillman MW (2008). Maternal smoking during pregnancy and child overweight: systematic review and meta-analysis. Int J Obes.

[44] Arenz S, Rückerl R, Koletzko B, von Kries R (2004). Breast-feeding and childhood obesity—a systematic review. Int J Obes.

[45] Seach KA, Dharmage SC, Lowe AJ, Dixon JB (2010). Delayed introduction of solid feeding reduces child overweight and obesity at 10 years. Int J Obes.

[46] Monteilh C, Kieszak S, Flanders WD, Maisonet M, Rubin C, Holmes AK (2011). Timing of maturation and predictors of Tanner stage transitions in boys enrolled in a contemporary British cohort. Paediatr Perinat Epidemiol.

[47] Christensen Krista Yorita, Maisonet Mildred, Rubin Carol, Holmes Adrianne, Flanders W. Dana, Heron Jon, Golding Jean, McGeehin Michael A., Marcus Michele (2010). Pubertal Pathways in Girls Enrolled in a Contemporary British Cohort. International Journal of Pediatrics.

[48] Neymotin F, Nemzer LR (2014). Locus of control and obesity. Front Endocrinol.

[49] Reilly JJ, Armstrong J, Dorosty AR, Emmett PM, Ness A, Rogers I (2005). Early life risk factors for obesity in childhood: cohort study. Br Med J.

[50] Timpson NJ, Emmett PM, Frayling TM, Rogers I, Hattersley AT, McCarthy MI, Smith GD (2008). The fat mass–and obesity-associated locus and dietary intake in children. Am J Clin Nutr.

[51] Gale CR, Batty GD, Deary IJ (2008). Locus of control at age 10 years and health outcomes and behaviors at age 30 years: the 1970 British Cohort Study. Psychosom Med.

[52] Fraser A., Macdonald-Wallis C., Tilling K., Boyd A., Golding J., Davey Smith G., Henderson J., Macleod J., Molloy L., Ness A., Ring S., Nelson S. M., Lawlor D. A. (2012). Cohort Profile: The Avon Longitudinal Study of Parents and Children: ALSPAC mothers cohort. International Journal of Epidemiology.

[53] Anzman SL, Rollins BY, Birch LL (2010). Parental influence on children’s early eating environments and obesity risk: implications for prevention. Int J Obes.

[54] Campis LK, Lyman RD, Prentice-Dunn S (1986). The parental locus of control scale: development and validation. J Clin Child Psychol.

[55] Wallston BS, Wallston KA (1978). Locus of control and health: a review of the literature. Health Educ Monogr.

[56] Hagekull B, Bohlin G, Hammerberg A (2001). The role of parental perceived control in child development: a longitudinal study. Int J Behav Dev.

[57] Moreland AD, Felton JW, Hanson RF, Jackson C, Dumas JE (2016). The relation between parenting stress, locus of control and child outcomes: predictors of change in a parenting intervention. J Child Fam Stud.

[58] Rouech J, Mink O, Roueche John, Mink Oscar (1976). Locus of control and success expectancy. Improving student motivation.

[59] Wolinsky F, Vander Weg MW, Martin R, Unverzagt FW, Willis SL, Marsiske M (2010). Does cognitive training improve internal locus of control among older adults. J Gerontol: Psychol Sci Soc Sci.

[60] Nowicki S (2016). Choice or chance.

[61] Hassall R, Rose J (2005). Parental cognitions and adaptation to the demands of caring for a child with an intellectual disability: a review of the literature and implications for clinical interventions. Behav Cogn Psychother.

