# Supplementary material for: The relationship between parental locus of control and adolescent obesity: a longitudinal pre-birth cohort
Source: Int J Obes (Lond). 2018 Jul 9;43(4):724–34. doi: 10.1038/s41366-018-0141-y (PMC6215477; doi:10.1038/s41366-018-0141-y)
Supplement: Supplementary file 1 — Supplementary Tables [file 41366_2018_141_MOESM1_ESM.docx]

**Supplementary Table 1.** The mean [SD] and 85^th^ centile of the measured fat mass in kg for the adolescents in the study.

| **AGE OF**  **ADOLESCENT** | **NO.**  **MEASURED** | **MEAN** | **SD** | **85^th^ CENTILE** |
| --- | --- | --- | --- | --- |
|  |  |  |  |  |
| 9 years | 7329 | 8.577 | 5.157 | 13.756 |
|  |  |  |  |  |
| 11 years | 7001 | 11.768 | 6.859 | 18.860 |
|  |  |  |  |  |
| 13 years | 6032 | 13.788 | 8.095 | 21.877 |
|  |  |  |  |  |
| 15 years | 5154 | 15.382 | 9.219 | 24.081 |
|  |  |  |  |  |
| 17 years | 4850 | 18.307 | 10.539 | 28.146 |

**Supplementary Table 2**. The raw data concerning the mean [SD] fat mass (kg) of the adolescents according to the locus of control orientation of the mother, the father and study child during pregnancy,

|  | **9 years** | **11 years** | **13 years** | **15 years** | **17 years** |
| --- | --- | --- | --- | --- | --- |
|  |  |  |  |  |  |
| **Maternal** |  |  |  |  |  |
| ELOC |  |  |  |  |  |
| Mean [sd] | 8.94 [5.50] | 12.26 [7.25] | 14.56 [8.74] | 16.39 [9.81] | 19.21 [11.31] |
| (n) | (2617) | (2463) | (2078) | (1692) | (1541) |
|  |  |  |  |  |  |
| ILOC |  |  |  |  |  |
| Mean[sd] | 8.24 [4.83] | 11.36 [6.52] | 13.19 [7.53] | 14.68 [8.61] | 17.63 [9.91] |
| (n) | (4091) | (3956) | (3487) | (3069) | (2869) |
|  |  |  |  |  |  |
| MD  [95%CI] | 0.71  [0.46, 0.96] | 0.91  [0.56, 1.25] | 1.36  [0.93, 1.80] | 1.70  [1.16, 2.24] | 1.57  [0.93, 2.22] |
| P | < 0.0001 | < 0.0001 | < 0.0001 | < 0.0001 | < 0.0001 |
|  |  |  |  |  |  |
| **Paternal** |  |  |  |  |  |
| ELOC |  |  |  |  |  |
| Mean [sd] | 8.83 [5.36] | 12.11 [7.20] | 14.35 [8.60] | 16.16 [9.87] | 19.06 [11.37] |
| (n) | (2100) | (2043) | (1736) | (1478) | (1347) |
|  |  |  |  |  |  |
| ILOC |  |  |  |  |  |
| Mean[sd] | 8.25 [4.82] | 11.28 [6.31] | 13.08 [7.33] | 14.67 [8.45] | 17.46 [9.65] |
| (n) | (2880) | (2760) | (2492) | (2180) | (2027) |
|  |  |  |  |  |  |
| MD  [95%CI] | 0.58  [0.29, 0.86] | 0.83  [0.45, 1.22] | 1.27  [0.78, 1.75] | 1.49  [0.89, 2.09] | 1.60  [0.88, 2.31] |
| P | < 0.0001 | < 0.0001 | < 0.0001 | < 0.0001 | < 0.0001 |
|  |  |  |  |  |  |
| **Childhood** |  |  |  |  |  |
| ELOC |  |  |  |  |  |
| Mean [sd] | 8.59 [5.23] | 11.97 [7.04] | 14.43 [8.49] | 16.14 [9.88] | 19.04 [11.00] |
| (n) | (2373) | (2232) | (1945) | (1663) | (1440) |
|  |  |  |  |  |  |
| ILOC |  |  |  |  |  |
| Mean[sd] | 8.43 [5.01] | 11.51 [6.65] | 13.28 [7.80] | 14.64 [8.69] | 17.28 [9.95] |
| (n) | (3588) | (3396) | (3021) | (2639) | (2372) |
|  |  |  |  |  |  |
| MD  [95%CI] | 0.16  [-0.10, 0.43] | 0.46  [0.10, 0.83] | 1.15  [0.69, 1.61] | 1.50  [0.93, 2.06] | 1.76  [1.09, 2.44] |
| P | 0.233 | 0.013 | < 0.0001 | < 0.0001 | < 0.0001 |
|  |  |  |  |  |  |

ELOC = external locus of control; ILOC = internal locus of control; MD = mean difference; CI = confidence interval.

**Supplementary Table 3**. Associations between **paternal** external locus of control as measured in pregnancy and fat mass in adolescence. The table shows the mean difference (kg) in fat mass for offspring of externally compared with internally oriented men.

| **AGE OF**  **OFFSPRING** | **MODEL A** | **MODEL B** | **MODEL C** |
| --- | --- | --- | --- |
|  |  |  |  |
| **9 years** |  |  |  |
| MD [95% CI] | 0.54 [0.26, 0.82] | 0.38 [0.09, 0.68] | 0.38 [0.05, 0.72] |
| P | < 0.001 | 0.010 | 0.025 |
| N | 4882 | 4661 | 3318 |
|  |  |  |  |
| **11 years** |  |  |  |
| MD [95% CI] | 0.82 [0.44, 1.20] | 0.55 [0.15, 0.95] | 0.68 [0.24, 1.11] |
| P | < 0.001 | 0.007 | 0.002 |
| N | 4704 | 4492 | 3545 |
|  |  |  |  |
| **13 years** |  |  |  |
| MD [95% CI] | 1.18 [0.72, 1.64] | 0.83 [0.35, 1.31] | 1.00 [0.47, 1.53] |
| P | < 0.001 | 0.001 | < 0.001 |
| N | 4151 | 3985 | 3091 |
|  |  |  |  |
| **15 years** |  |  |  |
| MD [95% CI] | 1.32 [0.77, 1.87] | 1.06 [0.49, 1.64] | 1.20 [0.57, 1.84] |
| P | < 0.001 | < 0.001 | < 0.001 |
| N | 3596 | 3457 | 2752 |
|  |  |  |  |
| **17 years** |  |  |  |
| MD [95% CI] | 1.54 [0.86, 2.21] | 1.14 [0.43, 1.84] | 1.16 [0.37, 1.94] |
| P | < 0.001 | 0.002 | 0.004 |
| N | 3315 | 3176 | 2433 |
|  |  |  |  |

Model A allows for sex and ethnic background of the child; model B additionally allows for duration of breast feeding, age started solids and whether the mother smoked at mid-pregnancy; Model C is model B plus aspects of the offspring diet at ages 10 (energy, trans fatty acids, sugar) and activity levels at 11.

MD = mean difference; CI = confidence interval.

**Supplementary Table 4**. Comparison between adjusted mean differences [95%CI] in fat mass of boys and girls, for children of **external mothers** compared with those of internal mothers.

| **AGE OF**  **OFFSPRING** | **BOYS MODEL**  **A** | **BOYS MODEL**  **C** | **GIRLS MODEL A** | **GIRLS MODEL**  **C** |
| --- | --- | --- | --- | --- |
|  |  |  |  |  |
| **9 years** |  |  |  |  |
| MD [95%CI] | 0.41 [0.07, 0.75] | 0.05 [-0.43, 0.54] | 0.92 [0.57, 1.27] | 0.69 [0.24, 1.15] * |
| P | 0.019 | 0.825 | <0.0001 | 0.003 |
| N | 3178 | 1439 | 3265 | 1801 |
|  |  |  |  |  |
| **11 years** |  |  |  |  |
| MD [95%CI] | 0.69 [0.21, 1.17] | 0.55 [-0.10, 1.20] | 1.09 [0.61, 1.56] | 0.74 [0.17, 1.31] |
| P | 0.005 | 0.099 | < 0.0001 | 0.011 |
| N | 3045 | 1538 | 3138 | 1910 |
|  |  |  |  |  |
| **13 years** |  |  |  |  |
| MD [95%CI] | 0.91 [0.32, 1.49] | 0.56[-0.22, 1.33] | 1.44 [0.87, 2.00] | 1.07[0.40, 1.74] |
| P | 0.002 | 0.157 | < 0.0001 | 0.002 |
| N | 2637 | 1370 | 2741 | 1703 |
|  |  |  |  |  |
| **15 years** |  |  |  |  |
| MD [95%CI] | 1.06 [0.36, 1.77] | 0.86 [-0.06, 1.79] | 1.52 [0.87, 2.18] | 1.18[0.39, 1.97] |
| P | 0.003 | 0.067 | < 0.0001 | 0.003 |
| N | 2188 | 1225 | 2420 | 1524 |
|  |  |  |  |  |
| **17 years** |  |  |  |  |
| MD [95%CI] | 0.82 [-0.08, 1.73] | 0.41 [-0.77, 1.60] | 1.62 [0.85, 2.39] | 0.81[-0.12, 1.74] |
| P | 0.074 | 0.492 | < 0.0001 | 0.086 |
| N | 1883 | 1015 | 2379 | 1413 |
|  |  |  |  |  |

Model A allows for sex and ethnic background of the child; model B additionally allows for duration of breast feeding, age started solids and whether the mother smoked at mid-pregnancy; Model C is model B plus aspects of the offspring diet at ages 10 (energy, trans fatty acids, sugar) and activity levels at 11.

MD = mean difference; CI = confidence interval.

*significant difference between boys and girls

**Supplementary Table 5**. Comparison between adjusted mean differences [95%CI] Kg in fat mass of boys and girls, for children of **external fathers**.

| **AGE OF**  **OFFSPRING** | **BOYS**  **MODEL**  **A** | **BOYS MODEL**  **C** | **GIRLS MODEL**  **A** | **GIRLS MODEL**  **C** |
| --- | --- | --- | --- | --- |
|  |  |  |  |  |
| **9 years** |  |  |  |  |
| MD[95%CI] | 0.49  [0.09, 0.89] | 0.23  [-0.32, 0.78] | 0.58  [0.19, 0.97] | 0.24  [-0.26, 0.75] |
| P | 0.015 | 0.415 | 0.004 | 0.349 |
| N | 2411 | 1156 | 2471 | 1412 |
|  |  |  |  |  |
| **11 years** |  |  |  |  |
| MD[95%CI] | 0.70  [0.15, 1.25] | 0.65  [-0.08, 1.39] | 0.93  [0.40, 1.45] | 0.43  [-0.22, 1.08] |
| P | 0.012 | 0.082 | 0.001 | 0.194 |
| N | 2314 | 1231 | 2390 | 1496 |
|  |  |  |  |  |
| **13 years** |  |  |  |  |
| MD[95%CI] | 1.15  [0.48, 1.82] | 0.98  [0.11, 1.86] | 1.19  [0.57, 1.82] | 0.85  [0.09, 1.61] |
| P | 0.001 | 0.028 | < 0.0001 | 0.028 |
| N | 2042 | 1106 | 2109 | 1343 |
|  |  |  |  |  |
| **15 years** |  |  |  |  |
| MD[95%CI] | 1.50  [0.68, 2.32] | 1.36  [0.30, 2.42] | 1.15  [0.41, 1.90] | 0.76  [-0.14, 1.66] |
| P | < 0.0001 | 0.012 | 0.002 | 0.096 |
| N | 1709 | 1005 | 1887 | 1228 |
|  |  |  |  |  |
| **17 years** |  |  |  |  |
| MD[95%CI] | 1.44  [0.40, 2.48] | 1.06  [-0.28, 2.41] | 1.62  [0.73, 2.50] | 0.94  [-0.15, 2.03] |
| P | 0.007 | 0.121 | < 0.0001 | 0.092 |
| N | 1470 | 836 | 1845 | 1131 |
|  |  |  |  |  |

Model A allows for sex and ethnic background of the child; model B additionally allows for duration of breast feeding, age started solids and whether the mother smoked at mid-pregnancy; Model C is model B plus aspects of the offspring diet at ages 10 (energy, trans fatty acids, sugar) and activity levels at 11.

MD = mean difference; CI = confidence interval.

**Supplementary Table 6**. Comparison between adjusted mean differences [95% CI] Kg in fat mass of adolescent boys and girls, if **their** LOC orientation at age 8 was external.

|  | **BOYS**  **MODEL A** | **BOYS**  **MODEL C** | **GIRLS**  **MODEL A** | **GIRLS**  **MODEL C** |
| --- | --- | --- | --- | --- |
|  |  |  |  |  |
| **9 years** |  |  |  |  |
| MD[95%CI] | 0.19  [-0.18, 0.56] | 0.24  [-0.29, 0.77] | 0.09  [-0.29, 0.47] | -0.13  [-0.60, 0.34] |
| P | 0.314 | 0.370 | 0.639 | 0.598 |
| N | 2686 | 1291 | 2777 | 1607 |
|  |  |  |  |  |
| **11 years** |  |  |  |  |
| MD[95%CI] | 0.44  [-0.09, 0.97] | 0.50  [-0.21, 1.21] | 0.47  [-0.04, 0.89] | -0.03  [-0.63, 0.57] |
| P | 0.105 | 0.170 | 0.071 | 0.926 |
| N |  | 1359 | 2630 | 1681 |
|  |  |  |  |  |
| **13 years** |  |  |  |  |
| MD[95%CI] | 1.30  [0.64, 1.95] | 0.80  [-0.05, 1.64] | 0.95  [0.34, 1.57] | 0.51  [-0.20, 1.22] |
| P | < 0.0001 | 0.064 | 0.002 | 0.162 |
| N | 2239 | 1226 | 2337 | 1525 |
|  |  |  |  |  |
| **15 years** |  |  |  |  |
| MD[95%CI] | 1.19  [0.41, 1.96] | 1.17  [0.16, 2.18] | 1.51  [0.80, 2.23] | 0.92  [0.09, 1.75] |
| P | 0.003 | 0.023 | < 0.0001 | 0.030 |
| N | 1904 | 1110 | 2071 | 1371 |
|  |  |  |  |  |
| **17 years** |  |  |  |  |
| MD[95%CI] | 1.59  [0.56, 2.62] | 1.13  [-0.18, 2.45] | 1.28  [0.44, 2.12] | 0.86  [-0.12,1.84] |
| P | 0.002 | 0.091 | 0.003 | 0.086 |
| N | 1595 | 919 | 1953 | 1265 |
|  |  |  |  |  |

Model A allows for sex and ethnic background of the child; model B additionally allows for duration of breast feeding, age started solids and whether the mother smoked at mid-pregnancy; Model C is model B plus aspects of the offspring diet at ages 10 (energy, trans fatty acids, sugar) and activity levels at 11.

MD = mean difference; CI = confidence interval.

**Supplementary Table 7**. Analyses of prediction of obesity (fat mass > 85^th^ centile) by each of the three external locus of control traits, both singly and in combination.

| **Age** | **Model** | **Maternal ELOC**  **OR [95% CI]** | **Paternal ELOC**  **OR [95% CI]** | **Child’s ELOC**  **OR [95% CI]** |
| --- | --- | --- | --- | --- |
|  |  |  |  |  |
| 9 |  | *Mother only* | *Father only* | *Child only* |
|  | A | 1.47[1.27,1.69]**** | 1.35[1.15,1.59]**** | 1.11[0.95,1.30] |
|  | B | 1.31[1.13,1.53]**** | 1.24[1.04,1.48]* | 1.06[0.90,1.24] |
|  | C | 1.41[1.17,1.70]**** | 1.32[1.07,1.63]* | 1.15[0.94,1.39] |
| 9 |  | *Mother + child* |  | *Mother + child* |
|  | A | 1.39[1.19,1.63]*** | - | 1.09[0.93,1.27] |
|  | B | 1.30[1.10,1.53]** | - | 1.04[0.88,1.22] |
|  | C | 1.37[1.12,1.67]** | - |  |
| 9 |  |  | *Father + child* | *Father + child* |
|  | A | - | 1.27[1.06,1.53]** | 1.08[0.90,1.30] |
|  | B | - | 1.25[1.03,1.51]* | 1.02[0.84,1.23] |
|  | C | - | 1.33[1.06,1.68]* | 1.12[0.89,1.40] |
|  |  |  |  |  |
| 11 |  | *Mother only* | *Father only* | *Child only* |
|  | A | 1.37[1.19,1.58]**** | 1.52[1.29,1.80]**** | 1.19[1.01,1.39]* |
|  | B | 1.25[1.07,1.46]** | 1.41[1.18,1.67]**** | 1.13[0.96,1.33] |
|  | C | 1.36[1.14,1.63]*** | 1.50[1.22,1.83]**** | 1.14[0.95,1.38] |
|  |  |  |  |  |
| 11 |  | *Mother + child* |  | *Mother + child* |
|  | A | 1.38[1.17,1.62]**** | - | 1.15[0.98,1.35] |
|  | B | 1.30[1.10,1.54]** | - | 1.10[0.93,1.30] |
|  | C | 1.34[1.10,1.63]** | - | 1.11[0.92, 1.34] |
| 11 |  |  | *Father + child* | *Father + child* |
|  | A | - | 1.54[1.28,1.86]**** | 1.08[0.89,1.30] |
|  | B | - | 1.48[1.22,1.79]**** | 1.00[0.83,1.22] |
|  | C | - | 1.57[1.26,1.96]**** | 1.00[0.80,1.25] |
|  |  |  |  |  |
| 13 |  | *Mother only* | *Father only* | *Child only* |
|  | A | 1.63[1.39,1.90]**** | 1.44[1.21,1.72]**** | 1.51[1.28,1.79]**** |
|  | B | 1.40[1.19,1.65]**** | 1.26[1.05,1.52]* | 1.42[1.19,1.69]**** |
|  | C | 1.42[1.17,1.71]**** | 1.30[1.05,1.61]* | 1.42[1.16,1.73]*** |
|  |  |  |  |  |
| 13 |  | *Mother + child* |  | *Mother + child* |
|  | A | 1.59[1.34,1.88]**** | - | 1.45[1.22,1.72]**** |
|  | B | 1.43[1.19,1.72]*** | - | 1.38[1.16,1.64]*** |
|  | C | 1.37[1.11,1.69]** | - | 1.39[1.13,1.69]*** |
| 13 |  |  | *Father + child* | *Father + child* |
|  | A | - | 1.39[1.14,1.69]** | 1.37[1.12,1.66]** |
|  | B | - | 1.23[1.00,1.51]* | 1.27[1.04,1.56]* |
|  | C | - | 1.29[1.02,1.63]* | 1.29[1.03,1.63]* |

**Supplementary Table 7 continued**

| **Age** | **Model** | **Maternal ELOC**  **OR [95% CI]** | **Paternal ELOC**  **OR [95% CI]** | **Child’s ELOC**  **OR [95% CI]** |
| --- | --- | --- | --- | --- |
|  |  |  |  |  |
| 15 |  | *Mother only* | *Father only* | *Child only* |
|  | A | 1.60[1.35,1.89]**** | 1.41[1.16,1.70]*** | 1.55[1.30,1.86]**** |
|  | B | 1.41[1.18,1.69]*** | 1.28[1.04,1.56]* | 1.45[1.20,1.75]**** |
|  | C | 1.32[1.07,1.62]** | 1.34[1.07,1.68]* | 1.41[1.14,1.74]** |
|  |  |  |  |  |
| 15 |  | *Mother + child* |  | *Mother + child* |
|  | A | 1.59[1.32,1.91]*** | - | 1.48[1.23,1.78]**** |
|  | B | 1.41[1.16,1.72]*** | - | 1.40[1.16,1.70]*** |
|  | C | 1.29[1.03,1.62]* | - | 1.37[1.11,1.70]** |
| 15 |  |  | *Father + child* | *Father + child* |
|  | A | - | 1.33[1.08,1.64]** | 1.38[1.12,1.70]** |
|  | B | - | 1.21[0.97,1.51] | 1.30[1.05,1.62]* |
|  | C | - | 1.26[0.98,1.61] | 1.31[1.03,1.67]* |
|  |  |  |  |  |
| 17 |  | *Mother only* | *Father only* | *Child only* |
|  | A | 1.47[1.23,1.75]**** | 1.41[1.16,1.72]*** | 1.49[1.23,1.81]**** |
|  | B | 1.27[1.06,1.53]* | 1.28[1.04,1.57]* | 1.46[1.20,1.79]**** |
|  | C | 1.27[1.02,1.59]* | 1.23[0.97,1.57] | 1.43[1.14,1.79]** |
|  |  |  |  |  |
| 17 |  | *Mother + child* |  | *Mother + child* |
|  | A | 1.34[1.10,1.64]** | - | 1.44[1.18,1.75]*** |
|  | B | 1.23[0.99,1.51] | - | 1.42[1.16[1.73]** |
|  | C | 1.16[0.91[1.48] | - | 1.39[1.11,1.74]** |
| 17 |  |  | *Father + child* | *Father + child* |
|  | A |  | 1.26[1.01,1.58]* | 1.37[1.09,1.71]** |
|  | B |  | 1.20[0.95,1.51] | 1.33[1.06,1.67]* |
|  | C |  | 1.13[0.87,1.48] | 1.34[1.04,1.74]* |
|  |  |  |  |  |

Model A allows for sex and ethnic background of the child; model B additionally allows for duration of breast feeding, age started solids and whether the mother smoked at mid-pregnancy; Model C is model B plus aspects of the offspring diet at ages 10 (energy, trans fatty acids, sugar) and activity levels at 11.

* P<0.05; ** P< 0.01; *** P< 0.001; **** P<0.0001
